# Modality-specific temporal constraints for state-dependent interval timing

**DOI:** 10.1038/s41598-018-28258-4

**Published:** 2018-07-03

**Authors:** Michele Fornaciai, Eleni Markouli, Massimiliano Di Luca

**Affiliations:** 1 Department of Psychological and Brain Sciences, University of Massachusetts, Amherst, MA USA; 2 Aristotle University of Thessaloniki, School of Philosophy, Department of Psychology, Thessaloniki, Greece; 3 Centre for Computational Neuroscience and Cognitive Robotics, School of Psychology, University of Birmingham, Edgbaston, Birmingham B15 2TT UK

## Abstract

The ability to discriminate temporal intervals in the milliseconds-to-seconds range has been accounted for by proposing that duration is encoded in the dynamic change of a neuronal network state. A critical limitation of such networks is that their activity cannot immediately return to the initial state, a restriction that could hinder the processing of intervals presented in rapid succession. Empirical evidence in the literature consistently shows impaired duration discrimination performance for 100 ms intervals demarked by short auditory stimuli immediately preceded by a similar interval. Here we tested whether a similar interference is present with longer intervals (300 ms) demarked either by auditory or by visual stimuli. Our results show that while temporal estimates of auditory stimuli in this range are not affected by the interval between them, duration discrimination with this duration is significantly impaired with visual intervals presented in rapid succession. The difference in performance between modalities is overall consistent with state-dependent temporal computations, as it suggests that the limits due to slow neuronal dynamics greatly depends on the sensory modality with which the intervals are demarked, in line with the idea of intrinsic, modality-specific neural mechanisms for interval timing.

## Introduction

Despite its pervasive role in the human physical and perceptual world, and its fundamental importance for many sensory and motor processes, the perception of time still remains one of the less known products of neural computation. Several theories have been proposed in the last few decades, concerning the possible mechanisms and the neural substrates responsible for the perception of duration of sensory events^1,2^. One of the most prominent and studied models of time perception is the “Clock-Counter” model or “Pacemaker-Accumulator”^3–6^, according to which the perceived passage of time is proportional to the number of pulses emitted by a pacemaker. This approach remains the most popular as its predictions have been verified experimentally^7,8^, despite lacking a clear evidence for its neural substrate^9^.

More recently, there has been a departure from this computationally-inspired approach in search of a mechanism for duration perception that is biologically plausible^2,8,10^. One of such approaches is the “State-Dependent Network” (SDN) model (e.g.^11^) which proposes that the dynamically changing activity of complex neural networks processing sensory information is related to interval duration. Neural network simulations have shown that the SDN model could account for numerous characteristics of human temporal processing performance^12–15^. Experimental evidence suggests that the processing of very brief durations is deeply rooted in the sensory processing mechanisms^16,17^.

Evidence supporting the involvement of state-dependent computations underlying time processing, comes from studies investigating specific predictions based on the expected dynamics of neuronal networks. For instance, Karmarkar & Buonomano^18^ reasoned that because the network state at the end of the interval should depend on the initial state of the network, duration discrimination should be worse if the network performing the discrimination is not properly reset. To change the initial state, the authors presented irrelevant interval distractors before the stimuli to be judged and found worse duration discrimination if the test interval was preceded by a distractor with a variable duration (henceforth referred to as “distractor” task). According to Karmarkar & Buonomano^18^ this result suggests that unpredictable initial activity leads to additional variability in the state of the network and hence to a degradation of the precision of temporal estimates. Subsequent studies however challenged the interpretation of these results. In a subsequent study by Spencer *et al*.^19^ employing the same distractor task used by Karmarkar & Buonomano^18^, the effect of the variable distractors has been found to disappear when either the duration of the distractor is different compared to the test interval or when the test interval is longer than originally tested (300 ms instead of 100 ms). While the lack of an effect could be interpreted as a limit in SDN dynamics, an alternative interpretation can be advanced at least for the longer intervals, as they might be tapping into a different temporal processing mechanism. As a second possibility, the lack of effect with longer intervals may suggest that performance impairment with variable distractors is due to attentional effects, such as the failure to shift attention to the test interval after the distractor, affecting only brief stimuli. A third possibility is that increasing the duration of the test interval might increase the variability of the encoded interval so that relatively small differences in the precision of temporal estimates may be masked by such increase in variability. Furthermore, also other evidence argues against an explanation of results from the distractor task based on the SDN framework. Indeed, Burr *et al*.^20^ further showed that variable distractors decrease performance even if they are presented after the test stimulus or in a different sensory modality. Such findings are at odd with an explanation based on the effect of the initial state of a dedicated network, since distractors presented after or in another modality than the test stimuli should have no influence.

Besides the effect of such variable context of temporal estimates, Karmarkar & Buonomano^18^ and Buonomano *et al*.^21^ tested another prediction of the SDN model: the effect of a pause between the intervals to be judged, which should be long enough to allow the resetting of the network prior to the delivery of the second interval. In these studies, the authors presented two intervals each marked by two sounds with either the same or different frequency and either with a short or a long pause between the two intervals to be compared. They reason that intervals bounded by sounds with the same frequency should be processed by the same network, while markers defined by different frequencies should be represented by the activity of different neurons^22,23^. This allowed Buonomano and colleagues^18,21^ to test whether a long pause after the first interval allows the network to return to the initial state and properly process the second interval. They found impaired discrimination performances only for intervals defined by the same frequency with a short pause between them – an impairment that appears to be specific for temporal judgments, since frequency discrimination is not affected by the length of the pause.

Previous studies^19^ seem to suggest that the effect of a variable context does not extend to longer auditory durations as the SDN predicts (although leaving open different alternative possibilities due to attentional effects or an increased variability of temporal estimates at longer durations; see above), thus it may be possible that all the interference effects predicted by the SDN theory could be limited to very short intervals. However, as the SDN model concerns the intrinsic properties of neuronal networks, the extent of interference effects should depend on the specific modality tested. For instance, while the auditory modality provides a very sharp representation of the temporal properties of sensory stimulation, the visual modality has a lower temporal resolution^24–27^. Thus, a possibility is that the temporal extent of the interferences predicted by the SDN model might depend on the specific sensory modality tested. Here we first test whether discrimination performance with relatively long intervals (300 ms) in the auditory modality changes in the same way as it does with short intervals (100 ms^18^) due to the length of the pause between them, or whether also this effect does not extend to longer intervals similarly to what has been shown by Spencer *et al*.^19^. Then, as different sensory modalities have different temporal dynamics and different temporal resolutions, we investigate whether the disrupting effect of a short pause between stimuli might be observed also with relatively long intervals marked by visual stimuli.

## Results

### Experiment 1 – Auditory Duration Discrimination

In the first experiment, we attempted to replicate the results reported by Karmarkar & Buonomano^18^ and Buonomano *et al*.^21^ showing the influence of the pause between intervals (inter-stimulus interval, ISI) on discrimination performance, but using longer intervals (300 ms vs. the original 100 ms). Furthermore, in the auditory sequence conditions, we investigated whether intervals that start and end with different markers affect duration discrimination performance and whether they are susceptible to the influence of the pause between them (ISI). By presenting the two intervals as either the same sequence of sound frequencies or an inverse sequence of sounds we can also evaluate whether the similarity between intervals can influence performance.

**Figure 1:**
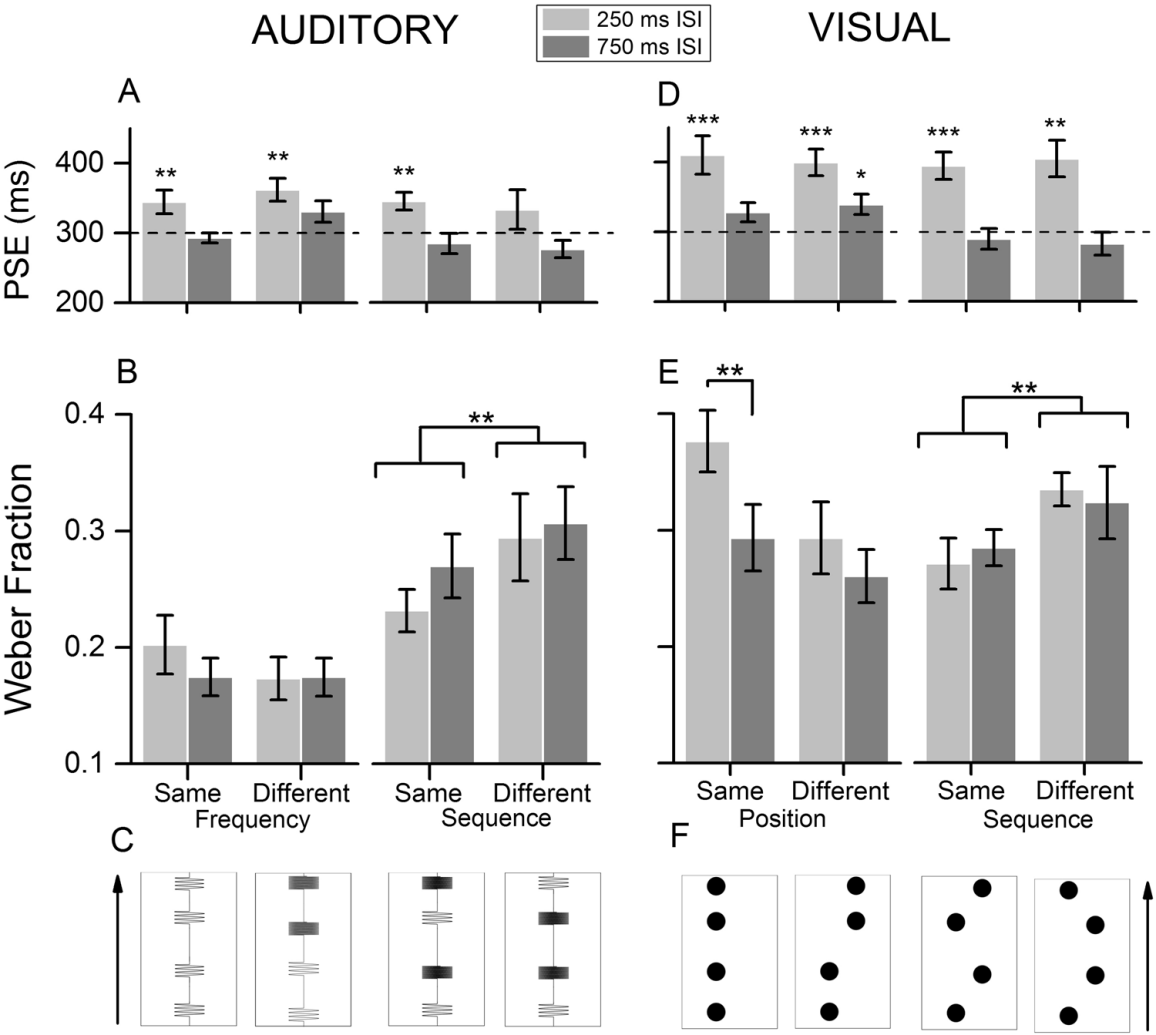
Results of Experiment 1 and 2. The two grey bars represent the results obtained with two different pauses between stimuli (ISI) for each condition. (**A**–**C**) Experiment 1. (**A**) Duration of the probe interval that perceptually matches the 300 ms standard (PSE). The dashed line marks the Point of Objective Equality (POE). (**B**) Average Weber Fraction (WF) in the auditory duration discrimination task. (**C**) Depiction of the auditory markers in the different conditions of Experiment 1. (**D**–**F**) Experiment 2. (**D**) Duration estimates (PSE). (**E**) Average WF in the two parts of Experiment 2. (**F**) Depiction of the experimental conditions in the visual experiment (not in scale). Note that the pictures are displayed in black and white, but the actual colors were red (background) and green (stimuli). Asterisks represent either the significance of one-sample t-tests against the physical standard duration (300 ms; panels A and D), or the significance of the multiple comparisons analysis (panels B and E). Error bars represent S.E.M. *p < 0.05, **p < 0.01, ***p < 0.001.

Figure 1A–C shows the results of Experiment 1. Regarding the duration estimates (PSE, i.e., duration of the second interval that appeared to match the first one; Fig. 1A), what we found is a systematic overestimation of the first interval, specifically with the 250 ms ISI, in three out of four conditions (one-sample t-tests against the physical reference duration [300 ms]: t(11) = 2.8, p = 0.019, t(11) = 3.9, p = 0.002 for the same and different frequency conditions, and t(11) = 3.5, p = 0.004 for the same sequence condition; no overestimation in the different sequence condition, t(11) = 1.1, p = 0.28). Conversely, with the 750 ms ISI duration estimates were almost veridical across all conditions (one-sample t-tests, all p-values > 0.05). To achieve a better index of the effect of ISI on the accuracy of temporal estimates, we then compared PSEs in the two ISI conditions against each other, instead of individually against the veridical duration of the reference intervals. The results of these tests showed that PSEs are significantly higher with the short ISI, in all the conditions tested (paired t-test, t(11) = 2.47, p = 0.015, t(11) = 2.62, p = 0.012, t(11) = 3.61, p = 0.002, t(11) = 2.05, p = 0.032, respectively for the same frequency, different frequency, same sequence, and different sequence condition).

Given that in several of the conditions tested we found a significant effect of ISI on perceived duration, we used Weber Fraction (WF) as a measure of participants’ precision (JND normalized by PSE). Such index takes into account the biases in perceived duration to give a more accurate measure of performance levels. What we found is reported in Fig. 1B. In the first part of Experiment 1, WF values were not significantly different neither comparing the different ISI, nor comparing the different conditions, (two-way R.M. ANOVA, F(1,11) = 0.89, p = 0.365, for factor “ISI”; F(1,11) = 2.3, p = 0.158 for factor “condition”), and no significant interaction emerged between the two factors (F(2,11) = 1.03, p = 0.331). In the auditory sequence conditions, on the other hand, we found a main effect of condition (two-way R.M. ANOVA, F(1,11) = 7.6, p = 0.018, *η*^2^ = 0.66) suggesting that the discrimination is made more difficult when the two intervals are marked by different sequences of tones. ISI had no systematic influence on performances also in this case (F(1,11) = 1.8, p = 0.205), and no interaction between the two factors was found (F(1,11) = 0.89, p = 0.36). A direct comparison between the overall performances in the two parts of the experiment confirmed that in the auditory sequence conditions of Exp. 1 WFs were significantly higher (Mann-Whitney Rank Sum test, U = 663, p = 0.001, Cohen’s d = 0.89), showing that comparing two intervals each defined by different markers is more difficult than comparing intervals marked by a similar sound frequency.

### Experiment 2 – Visual Duration Discrimination

Auditory and visual modalities have been shown to have different temporal resolution^24–27^. Because of these differences, in the second experiment, we tested whether modulating the ISI has a similar influence on the discrimination of visual intervals as it does on the auditory intervals^18^. We presented intervals marked by Gaussian blobs whose luminance was perceptually equated to the background to minimize apparent motion. To manipulate whether the two durations are processed by the same or by two different networks, here we presented stimuli either at one or two spatial locations, thus capitalizing on the segregation of visual receptive fields^28^ and localized processing of temporal properties revealed by spatially selective adaptation^29–31^. The second part of the experiment follows the logic of the auditory sequence conditions of Experiment 1: we presented the location of the markers either with a similar or a different sequence, which also served as a control for local interactions between the middle markers and for masking effects (see^32^ for a review).

Figure 1D–F shows the results of the two conditions of Experiment 2. First, regarding PSEs (Fig. 1D), we found a pattern of results very similar to the one obtained in the auditory experiment: in all the conditions with the short ISI, participants systematically overestimated the first interval (one-sample t-test against the physical reference duration of 300 ms; same position: t(11) = 4.213, p = 0.001; different position: t(11) = 5.5, p < 0.001, t(11) = 5.1; same sequence: p < 0.001; different sequence: t(11) = 4.1, p = 0.001). We also found a significant overestimation of the first interval in one of the conditions with the long ISI (different position: t(11) = 2.8, p = 0.015), while no differences were evident in the same position condition, t(11) = 2.1, p = 0.057, and in both the conditions of the visual sequences part of the experiment, t(11) = −0.73, p = 0.479 and t(11) = 1.1, p = 0.294). Comparing PSE estimates obtained in the two ISI conditions, on the other hand, confirmed that PSEs are significantly higher when intervals are separated by a short ISI, in all the conditions tested (paired t-test, t(11) = 2.72, p = 0.010, t(11) = 3.59, p = 0.002, t(11) = 5.36, p = 0.001, t(11) = 4.82, p = 0.001, respectively for the same position, different position, same sequence, and different sequence condition).

As in Experiment 1, since we found robust effects on PSE values, we used Weber Fraction as measure of precision (Fig. 1E). Regarding the first part of the experiment, a two-way R.M, ANOVA showed a significant main effect for both the factors, ISI (250 versus 750 ms; F(1,11) = 7.4, p = 0.02, *η*^2^ = 0.09) and position of the two intervals (same versus different; F(1,11) = 6.6, p = 0.026, *η*^2^ = 0.09), with no interaction between them (F(1,11) = 2.71, p = 0.13). A Holm-Sidak multiple comparison procedure further showed that WF were significantly higher with a 250 ms ISI, compared to the 750 ms ISI, but only in the same position condition (same position: t(11) = 3.166, p = 0.005, Cohen’s d = 0.91; different position: t(11) = 1.2, p = 0.22). In the visual sequence conditions, similar to Experiment 1, we found only a main effect of condition (same sequence versus different sequence), indicating that while ISI did not affect duration discrimination performances (F(1,11) = 0.004, p = 0.95), comparing intervals marked by a different sequence of visual stimuli was overall significantly more difficult (F(1,11) = 7.1, p = 0.022, *η*^2^ = 0.12). No interaction was observed between the two factors (F(1,11) = 0.603, p = 0.45). Finally, a direct comparison of the overall performances in the two parts of the experiment showed that WFs do not significantly differ as function of whether each interval was marked by stimuli in the same position or by two stimuli presented in different positions (Mann-Whitney Rank Sum test, U = 1120, p = 0.82), suggesting that the similarity of visual marker’s position has a much smaller influence compared to the sounds’ frequency for auditory intervals.

### Experiment 3 – Control: Size Discrimination

According to Karmarkar & Buonomano^18^, the effect of ISI should be specific to time perception. To investigate this specificity, we tested whether the decreased performance with short ISI generalizes to other perceptual discrimination tasks. To keep stimuli as similar as possible to the ones used in Experiment 2, we exploited a size discrimination task, using the same visual stimuli and presenting only the two middle markers.

**Figure 2:**
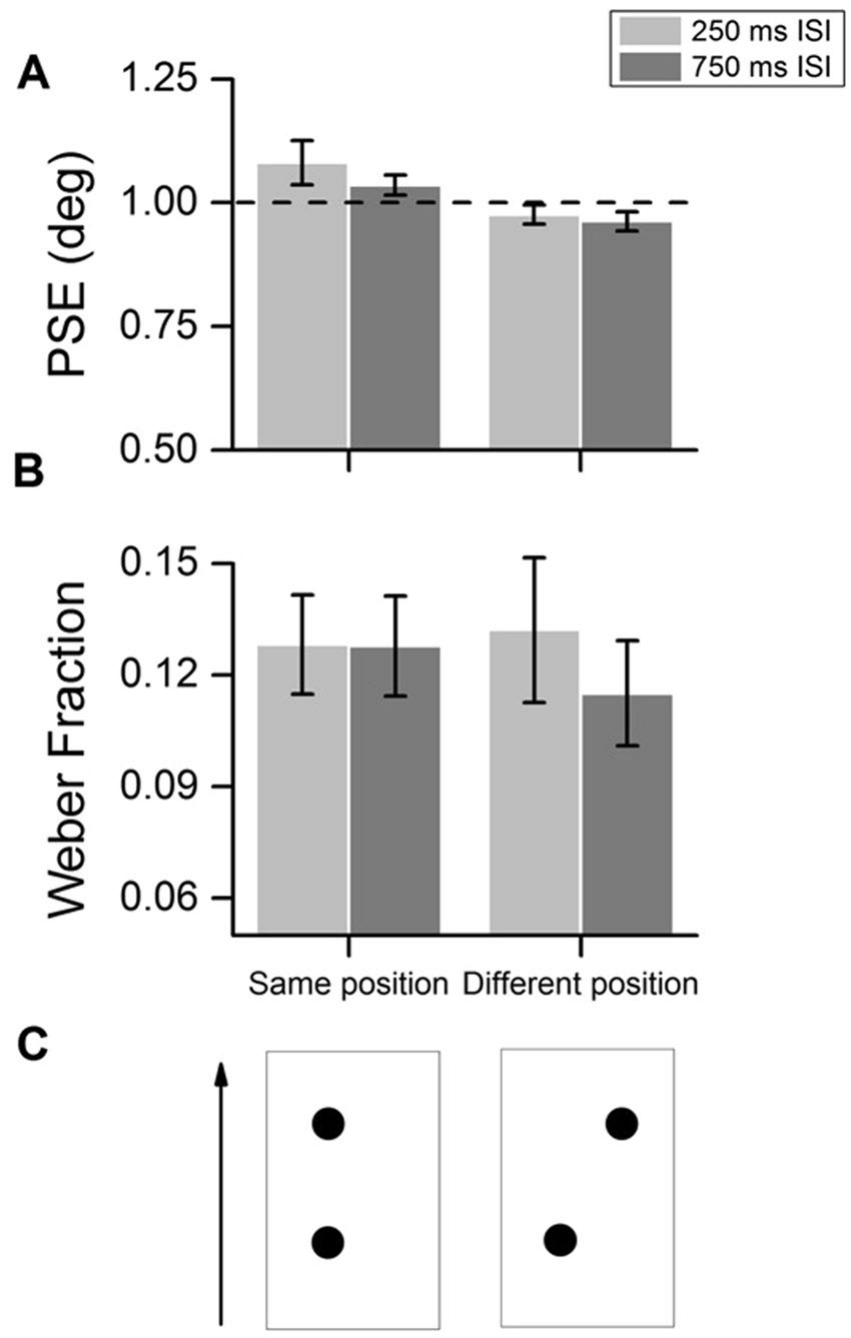
Results of Experiment 3 concerning visual size discrimination. Participants’ average PSE (**A**) and WF (**B**) in the two conditions of Experiment 3. (**C**) Schematic depiction of the two conditions (not in scale). Note that in the actual experiment stimuli were presented in green on a red background. Error bars represent S.E.M.

Figure 2 shows the results of the size discrimination experiment. Regarding participants’ perceived size (Fig. 2A), we did not observe any significant deviation from the veridical reference size (one-sample t-test against the physical reference size [1 deg]; same position condition, 250 ms ISI: t(7) = 1,9, p = 0.09, 750 ms ISI: t(7) = 1,8, p = 0.1; different position condition, 250 ms ISI: t(7) = −1.3, p = 0.22, 750 ms ISI: t(7) = −2.05, p .00.08). Crucially, we did not observe any significant effect also on participants’ WFs (Fig. 2B; two-way R.M. ANOVA; no differences neither in the factor ISI, F(1,7) = 1.1, p = 0.33, nor in the factor condition, F(1,7) = 1.4, p = 0.27), and no interaction between ISI and condition (F(1,7) = 0.23, p = 0.65). To achieve a better index of whether this null result reflects a robust evidence or just a lack of sensitivity (i.e., too few participants), we calculated the Bayes factor^33^ for the comparison between WF corresponding to short and long ISI in the same position condition. To do so, we hypothesized a lower bound for the difference in WF between conditions equal to zero (i.e., no effect of ISI), while as an upper bound of the ISI effect we took the average difference in WF between short and long ISI in the same position condition of Exp. 2 (0.083 ± 0.023), resulting in a Bayes factor equal to 0.24. Such low Bayes factor allows to conclude that the results obtained in this condition reflect a genuine lack of influence of the ISI on size discrimination performances.

## Discussion

While the clock-counter model^4,6^ probably remains the most prominent framework in time perception research, recent theories have suggested the possibility that the perception of time might be deeply rooted into sensory processing, proposing biologically-plausible mechanisms^8,34,35^. The predictions of one of these theoretical frameworks, the state-dependent network (SDN) model, have been tested directly by investigating contextual effects on discrimination performances, such as the effect of the inter-stimulus interval (ISI) between two empty intervals marked by short auditory stimuli^18,21^. Precision in duration discrimination tasks depends on the length of the pause between the two stimuli (ISI), supporting the idea that temporal information might be extracted from the state of the neuronal networks, which needs a proper “reset” between a new interval could be correctly processed.

However, other investigations further suggested a strict limitation of the SDN model, at least in the auditory modality, to very brief durations. Namely, Spencer and colleagues^19^, investigating the disruptive contextual effect on duration discrimination performances provided by distractor stimuli – previously demonstrated also by Karmarkar & Buonomano^18^ – found that such effect is no longer apparent when the base duration of the intervals is increased from 100 ms to 300 ms. While the effect found with the “distractor task” in Karmarkar & Buonomano’s^18^ paper provided intriguing evidence supporting their framework, the following results^19^ constrained the model to be limited to very brief durations.

Nevertheless, an intriguing possibility suggested by the SDN model is that neuronal networks presenting intrinsically different temporal dynamics might exploit state-dependent computations to represent time over different duration ranges. Here we attempted to study the contextual-dependency of human duration perception and the limits of the State-Dependent Network Model, investigating the impairing effect of a too short ISI on duration discrimination. To do so, we examined the ISI effect by presenting longer intervals compared to previous studies (300 ms), delivered by stimuli in different modalities (either auditory or visual). This paradigm has two main advantages compared to the “distractor” task, in terms of the inferences that can be made about the limits of state-dependent computations: first, data is less open to different interpretations than the one obtained with the “distractor” task (i.e., distractor and target interval tapping into different timing mechanisms); second, this task is less prone to attentional capture caused by the presentation of an irrelevant stimulus before the target interval, which may make the interpretation of the results more difficult (i.e., see^19^). In other words, although in principle the effect of changes in the frequency of auditory stimuli, of the position of visual stimuli, and of the different sensory modalities could be tested also using the distractor task, we reason that the interval discrimination is more robust to alternative interpretations, making it a more suitable paradigm to investigate the predictions of the SDN theory. Thus, if the lack of effect found by Spencer and colleagues^19^ reflects a feature of the SDN theory (i.e., state-dependent computations limited to very short durations), we expected to find similar temporal limits in the auditory modality in the interval comparison task – i.e., no effect using relatively long durations (300 ms). On the other hand, we reason that the range of durations that a neuronal network could be able to represent should be a function of the spectral content of sensory signals. Signals containing only low-frequency components should make the network change state more slowly than signals that include high-frequency content. Thus, since visual modality has a slower dynamics and lower temporal resolution than the auditory modality^24–27^, we expect that for visual signals the interference might extend beyond the previously observed temporal limits. In fact, Burr and colleagues^36^ have indeed shown that temporal properties could be captured by exponentially low-pass filtering the input signals and that the temporal characteristics of the filter are different across modalities, with a higher cutoff for audio than visual. Since, according to Karmarkar & Buonomano^18^, the reset time of the network should be a function of the longest time constant of the network properties represented, the visual modality should have a slower dynamics.

Results from Experiment 1, where we tested auditory duration discrimination, were consistent with Spencer *et al*.’s study^19^, suggesting that increasing the base duration to 300 ms attenuated the effect of ISI on performance. Here, we did not find a significant impairment in performance, neither with intervals bounded by sounds with the same frequency or with different frequencies. While these results do not ultimately challenge the SDN model, they suggest that the machinery for time processing proposed by the SDN model might be exploited only for very brief intervals.

Similarly, in the second part of Experiment 1 (auditory sequence conditions), we did not observe a systematic influence of ISI on duration discrimination performances, but only worse performance in the different frequency condition. This result suggests that sequences with mixed features might be less prone to contextual effect, but comparing intervals defined by markedly different features (i.e., opposite sequences) is overall more difficult – an effect that has been previously demonstrated by Horr & Di Luca^37^.

On the other hand, in the first part of Experiment 2, we found a markedly different result. In this experiment, to change whether the very same network or two different networks would be processing each of the two durations, we presented the visual intervals’ markers in either the same or two different positions. The spatial manipulation was chosen because visual neurons – from early to high-level stages – process information through spatially-localized receptive fields (e.g.^28,38^) and visual timing mechanisms are spatially localized^29–31^. What we found is that discrimination performance was impaired more by a short ISI than by the longer one, but only when the markers of the two intervals occupied the same positions in space. This selective impairment is consistent with the idea that sensory networks processing both intervals need a time to “reset” between two intervals. Importantly, we found that the impairment was present even though the base interval was 300 ms long, a duration that has been found to be free from such influence in the auditory modality.

As in the auditory conditions of Experiment 1, in the visual sequence conditions of Experiment 2 we did not observe any effect strictly related to the ISI, but we instead found a general decrease in temporal sensitivity for stimuli defined by markers presented according to different sequences. This effect seems related to a modality-independent difficulty in the comparison of intervals with different features^37^. Additionally, these results also rule out the influence of local interactions and masking effects between the last marker of the first interval and the first marker of the second interval (e.g.^32^).

Since we used just two different ISI conditions (250 ms and 750 ms), we could not determine how the results would look like at intermediate ISI durations. Two different patterns of results may be expected. A first possibility is that the disruptive effect of a too short interval should be proportional to the ISI duration, at least in the visual modality where we observed significant effects even with relatively long interval durations. What we may expect according to this idea, then, is a gradual increase in performance with longer ISIs. A second possibility is that the disruptive effect is only present if the ISI falls within the reset time needed for the network to obtain precise temporal estimates. In such a case, we may expect to find identical disruptive effects with multiple ISI duration, but no decrease in performance at all outside this range (i.e., a sudden switch from one level of ISI to a slightly longer one). We can speculate that the intrinsically variable dynamics of neural networks makes it less plausible to hypothesize a dichotomic effect. We thus predict that the effect should be inversely related to the ISI duration, gradually decreasing as the ISI gets longer. Testing this prediction could further the understanding of the SDN model.

How do the present results in the auditory modality compare with previous findings with the distractor task^19^? First, it should be noted that our ISI range is different from the range of interval durations used by Spencer and colleagues^19^. Indeed, our “short” ISI (250 ms) overlaps with the “long” variable interval range used by Spencer and colleagues^19^ (around 300 ms), and, consistently with this, we did not observe effects in the auditory modality with our “short” ISI. Overall, then, it seems that the lack of effect found with a relatively long duration (as a function of sensory modality) parallels previous results, although there might be a few key differences. First, when considering the boundaries of state-dependent computations investigated in the present and previous studies, it might be interesting to compare the ISI used here and the distractor interval used in previous studies^19^. Such a comparison, however, is not straightforward because the length of the distractor interval in Spencer *et al*.’s^19^ study could be compared with either the base duration of the intervals in the task used in the present study (300 ms) or with the ISI itself. Because participants tested with the distractor task^19^ were not aware of whether the first stimulus presented was marking the start of a target interval or the start of a distractor interval, we may interpret the distractor interval as a proper duration being encoded in the network’s dynamic, with ISI in this case being zero. By assuming this, it seems that increasing the duration of the interval makes the effect of ISI to disappear completely, thus suggesting that once outside the temporal range of state-dependent computations no contextual effect should take place (i.e., intervals in different ranges do not interact with each other). This prediction is in line with our findings. On the other hand, if the distractor interval in the distractor task is akin to the ISI in the interval task, our results in the auditory modality should be comparable with previous studies^19^. Indeed, we observed no significant disruptive effect of a 250-ms ISI, similarly to Spencer *et al*.’s^19^ results showing no consistent effect of a 300-ms distractor interval. Irrespective of which interpretation can be given of the distractor interval, our results are in line with previous ones despite using a different task, and they suggest that no disruptive contextual effect are present outside the boundaries of state-dependent computations.

The crucial question raised by our results is then: why does a too short ISI impair visual duration discrimination even with a relatively long duration range while this effect is absent in auditory duration discrimination tasks? The explanation might reside in the sensory processing characteristics of vision. While auditory signals normally carry temporal information with a finer resolution, visual signals are processed with a relatively more sluggish dynamics that makes the visual modality normally less sensitive to the temporal dimension – a feature that has been clearly demonstrated by the temporal ventriloquist effect^25,26^. Hartcher-O’Brien *et al*.^39^, however, have shown that the inferiority of visual duration judgments could be reversed by providing stimuli with low signal-to-noise ratio, making auditory duration judgments equally precise or even worse than visual ones. Such reversal shows that limitation in precision of temporal estimates depends on the noise of the output of sensory processing, not on an intrinsic resolution of the sensory modality considered. In light of this finding, the differential effect of ISI on auditory and visual stimuli suggests that the limits of temporal processing with normal signals depend on the noise of the sensory stimulus and, critically in normal stimulation conditions, by the dynamics of sensory processing itself, which in the SDN model is the speed with which the activation in the neuronal network changes as a function of the input. Higher noise leads to sluggish responses and in turn to a better discrimination for long durations, whereas less noise leads to rapid activation changes making the representation more prone to cross talk and thus more limited in the range of representable durations. Following this logic, further increasing the duration of visual intervals should make the interference effect to disappear, in the same way that it happens with longer auditory intervals.

Alternative explanations for the effects found in the visual modality appear to be less suitable to explain our data. For instance, other processes that might have been involved in our study are Inhibition of Return (IOR; see^40^ for a review) and the negative cueing effect provided by transient attention^41,42^. However, both effects cannot explain our results. On the one hand, previous studies show that the IOR has a markedly slow time-course in discrimination tasks (compared to reaction time tasks), and hence it should have affected also intervals separated by a long ISI^43^; on the other hand, the disruptive effect of transient attention on temporal processing is consistently weaker when stimuli are isoluminant with the background^44^. Moreover, according to a recent study by Cicchini & Morrone^45^, while attention might modulate perceived time, precision in a duration discrimination task does not systematically depend on it. So, whilst Spencer and colleagues^19^ reported that attentional effects might alternatively explain their findings, this possibility seems less suitable for our results.

In addition to the effect of ISI on discrimination performances, we also found a robust and systematic effect on perceived duration (PSE) in almost all the auditory and visual conditions, which was not previously reported. While the SDN theory provides clear predictions concerning the effect of a too short ISI on precision, predictions concerning the accuracy of duration estimates are less clear. A possible explanation is that a short interval between the two durations might have caused a leak from one interval to the other, with part of the second interval being assimilated to the first one. Such a phenomenon would result in an overestimation of the first interval and an underestimation of the second one, which seems to be consistent with the effect on PSE observed across the auditory and visual conditions. An alternative explanation concerns the possibility that the pattern of results might be due to the specific stimulus presentation procedure employed (we always presented the reference interval first), easily inducing systematic *positive* (overestimation of the first interval) time-order effects^46^. This would be consistent with previous studies on time-order effects, showing that the biases we found are usually more pronounced with short ISIs, short durations and relatively weak stimuli^47–51^. Since we cannot discriminate between these two interpretations, we cannot conclude that interference effects are the only limiting factor for the accuracy (i.e., perceived duration) of temporal estimates.

Finally, our control condition concerning the non-temporal dimension of size showed that the ISI effect is a peculiar feature of time perception, since no impairment has been observed in the visual size discrimination task (Experiment 3). This result is consistent with the findings of Karmarkar & Buonomano^18^ in the auditory domain, which showed that ISI does not influence frequency discrimination performance. However, caution is in order when interpreting a null effect from such a different task. Although it is in line with our interpretation, the lack of a significant difference in the size discrimination task cannot be taken as conclusive evidence for the specificity of the influence of ISI on temporal judgments. It is left to future studies to investigate the specificity of the interference predicted by the SDN theory across temporal and non-temporal tasks.

## Conclusion

Overall, our results are consistent with the predictions of the State-Dependent Network model, which proposes that intrinsic processing of temporal information happens directly at the level of sensory processing. Intriguingly, while we provided other evidence that the effects predicted by SDN model are limited to very short durations in the auditory modality, we also showed that in the visual modality the effect of a too short interval could be observed at longer durations, suggesting that the constraints of the SDN model depend on the specific temporal uncertainty of signals and on the dynamics of sensory processing.

## Methods

### Subjects

Twelve subjects took part in Experiment 1 (8 females, age range: 19–30 years), twelve subjects participated in Experiment 2 (9 females, age range 19–29 years), and eight subjects participated in Experiment 3 (5 females, age range 18–29 years) after giving informed written consent. With the exception of two of the authors (M.F. and E.M. which participated in all experiments), all other subjects were naive to purpose of the study and were rewarded with 6 GBP/hour. All participants had normal or corrected-to-normal vision and reported to have a good hearing. Experimental procedures were conducted according to the protocol approved by the STEM ethics committee of the University of Birmingham, and are in line with the declaration of Helsinki.

### Apparatus

The experiment was performed in a quiet dark room. Observers sat at a table, with head stabilized by means of a chinrest. The experiment comprised trials with two empty intervals separated by a pause. Each interval was delimited by two markers, one at the onset and one at the offset. Auditory (Experiment 1) and visual (Experiments 2 and 3) stimuli were used to mark the intervals. All stimuli were generated with the Psychophysics Toolbox^52–54^ for MatLab (MatLab 2013b, The Mathworks, Inc.). Auditory markers were 16 ms (2 ms onset and offset ramp) tones (~65 dB), either with 1 kHz or 4 kHz frequency delivered by a speaker (Fostex PM0.4n) positioned on the table at 60 cm in front of the participant. Visual markers were green Gaussian blobs (sigma = 1 deg), presented for one monitor frame (16,6 ms) on a red background (luminance = 13.44 cd/m^2^). The luminance of the green stimuli was chosen specifically for each subject according to the their perceived equiluminance. Perceived equiluminance was determined before each experimental session by means of a flicker-fusion procedure^55^. The green channel luminance in the main experiment was then adjusted according to the value that corresponded to the minimum impression of flicker (average matched luminance = 16.35 cd/m^2^). Stimuli were displayed in two possible positions: 5 deg to the left or to the right of the central fixation point (with 6 deg vertical eccentricity) and presented on a NEC Multisync CRT monitor, with a resolution of 800 × 600 and a refresh rate of 60 Hz, positioned 60 cm in front of the participant. Responses were collected using a standard keyboard.

### Procedure

#### Experiment 1

In each trial, two intervals were presented, each marked by two auditory stimuli. Subjects were instructed to indicate which one of the two intervals seemed to have a longer duration by pressing the appropriate key on a keyboard. The standard interval had a duration of 300 ms and was always presented first. The probe interval ranged between 150 ms and 550 ms in duration in 10 steps. The two intervals were separated by a pause (henceforth termed ISI for Inter Stimulus Interval) of either 250 or 750 ms (the same used in Karmarkar & Buonomano’s study^18^). Both ISI conditions were randomly interleaved within each block. In the two parts of the experiment, the different ISI conditions were combined with other two different conditions in a 2 × 2 experimental design. In the first part of the experiment, while the standard interval was always defined by two 1 kHz tones, the second interval could be marked either by 1 kHz (“same frequency” condition) or 4 kHz (“different frequency” condition) tones, with these two possibilities interleaved within each block. In the second part of the experiment (auditory “sequence” conditions), while the standard interval was defined by two markers having different frequencies (1 kHz at the onset and 4 kHz at the offset), the markers of the second interval could have either the two frequencies presented with the same order (1 kHz onset/4 kHz offset, “same sequence” condition) or with the opposite order (4 kHz onset/1 kHz offset; “different sequence” condition). Each participant completed four blocks of 80 trials for each part of the experiment. Figure 1C shows a depiction of the auditory conditions tested in Exp. 1.

#### Experiment 2

Each trial consisted in the presentation of a sequence of two intervals, each marked by two visual stimuli, while participants kept fixation on a small black circle at the center of the screen. Participants were instructed to indicate, at the end of each trial, which interval seemed longer, pressing the appropriate key on a keyboard. The first interval (standard) was kept constant, with a duration of 300 ms, while the second one was varied from trial to trial, with durations ranging from 150 to 550 ms. As in the Experiment 1, we tested two pauses between stimuli: 250 and 750 ms. In the first part of Experiment 2, we presented the two intervals either in the same or in different position, according to a 2 × 2 design (pauses and positions). All the combinations were randomly interleaved across each experimental session. In the second part of the experiment (visual “sequence” conditions), each of the two durations was bounded by flashes presented in two different locations. The positions of the first two markers were kept always the same across each experimental session, while the markers of the second interval could be presented as either the same sequence of flashes as the first interval, or as an inverse sequence. The two different pauses and the two different sequences were interleaved again according to a 2 × 2 design. Each subject completed four blocks of 80 trials for each part of the experiment. Figure 1F shows a summary of the different conditions (positions and sequences) tested in Experiment 2.

#### Experiment 3

Experiment 3 is a control for Experiment 2, so the conditions and procedure were kept similar to that of the first part of Experiment 2, except that in this case only two stimuli were presented (the two middle markers). Participants were instructed to indicate, at the end of each trial, which stimulus seemed bigger in size (not which one was longer as Experiment 1 and 2), by pressing the appropriate key on a keyboard. The size of the first stimulus (reference) was kept constant across the trials, with a width of the Gaussian envelope fixed at 1 deg, while the second one (probe) was modulated in size from trial to trial, with width varying from 0.51 deg to 1.51 deg. Stimuli could be presented either in the same or in different positions (with the first one always on the left of the fixation point), and separated by either a 250 or 750 ms pause. Each subject completed four blocks of 88 trials. The two conditions of Exp. 3 are depicted in Fig. 2C.

### Behavioral data analysis

Subjects’ accuracy and precision in the task were obtained by fitting a cumulative Gaussian function to the proportion of responses as function of the test duration, according to the maximum likelihood method^56^. As a measure of accuracy, we calculated the point of subjective equality (PSE), defined as the median of the best-fitting cumulative Gaussian curve to the data of a given condition. The PSE provides a measure of the perceived duration of the standard stimulus under different stimulation conditions, as it represents the test duration perceptually matching (indistinguishable from) the standard stimulus. As a measure of precision in the task, we calculated the Weber fraction, defined according to the Weber-Fechner law as the minimum discriminable increment (just noticeable difference [JND]; the standard deviation of the best-fitting Gaussian function), normalized by the perceived duration of the standard stimulus (PSE). The same procedure was employed to obtain accuracy and precision measures of size estimates in Exp. 3. PSEs at the group level were tested with one-sample t-tests against the null hypothesis of no difference from the physical duration of the standard stimulus (300 ms in Exp. 1–2, 1 deg in Exp. 3). WFs across multiple conditions were tested using repeated measure ANOVAs. When comparing precision measures across different parts of an experiment (Exp. 1–2), we instead used Mann-Whitney Sum Rank tests, as pooling data from multiple conditions resulted in a failing of normality tests. Additionally, since in Experiment 3 we tested a smaller group of subjects, to ensure that the data obtained with this sample size was strong enough to draw robust conclusions, we calculated the Bayes factor^33^. This index indicates how strongly the data supports the null hypothesis (i.e., no effect of the experimental manipulation) or the alternative hypothesis (i.e., the experimental manipulation causes a significant effect), and allows to discriminate whether a null result is actually a strong evidence in favor of the null hypothesis or just a lack of evidence for both hypotheses. Across all the experiments, participants’ performance in each block of trials was checked to ensure a sufficient level of performance in the task. Three blocks of trials were excluded from data analysis due to poor performance, one in the first part of Experiment 1, one in the first part of Experiment 2, and one in Experiment 3.

### Data availability

The datasets generated during the experiments described in the manuscript will be provided by the corresponding author upon reasonable request.
